# The dilemma of ischemia testing with different methods

**DOI:** 10.1530/ERP-14-0013

**Published:** 2014-05-29

**Authors:** Maria Pia Donataccio, Claudio Reverberi, Nicola Gaibazzi

**Affiliations:** 1 Parma University Hospital, Parma, Italy; 2 Verona University Hospital, Verona, Italy

## Abstract

A 52-year-old man presented after one episode of effort angina, normal treadmill electrocardiogram (ECG), and clearly positive adenosine cardiac magnetic resonance (aCMR) for reversible perfusion defects in the left anterior descending (LAD) coronary artery territory. Contrast high-dose dipyridamole (0.84 mg/kg per 6 min) stress echocardiography (cSE) demonstrated normal myocardial perfusion (MP) and wall motion at rest, while perfusion defects were shown in the lateral and apical segments after dipyridamole. Wall motion at stress was completely normal and stress/rest Doppler diastolic velocity ratio on the LAD demonstrated reduced flow reserve. In this case, cSE was the provocative test detecting both the LAD and circumflex obstructive lesions, thanks to MP analysis, while wall motion assessment was negative, not different from treadmill ECG, and aCMR highlighted only the LAD disease.

**Learning points:**

- In spite of the low sensitivity of wall motion assessment during stress-echocardiography to detect coronary artery disease (CAD) in patients with multivessel disease and balanced ischemia, the addition of cSE with myocardial perfusion assessment, is not only able to overcome this limitation of false negative rate on a per-patient basis, but may also depict multivessel myocardial perfusion defects more efficiently than aCMR, as in the reported case, thanks to high spatial resolution.
- Myocardial perfusion assessment during cSE, although not always technically feasible, has a very high spatial and temporal resolution which can easily demonstrate multivessel subendocardial perfusion defects during maximal vasodilation, which is often the only detectable marker of multivessel, balanced CAD.
- It is known that wall motion analysis during pharmacologic stress may result in falsely negative multivessel disease; in these cases perfusion imaging or Doppler measurement of coronary flow reserve may be helpful to detect multivessel obstructive CAD, which is a significant and dismal prognostic finding. aCMR is assumed as the perfect imaging modality for CAD detection, but in selected cases, such as the one presented, an advanced echocardiographic method in experienced hands can provide even more comprehensive results.

## Background

The combination of myocardial perfusion (MP) imaging and dipyridamole or dobutamine real-time contrast echocardiography improves the sensitivity to detect coronary artery disease (CAD), particularly multivessel CAD. Patients with diffused CAD have the worst prognosis and hence false-negative results represent Achilles heel of stress echocardiography, when performed in its standard fashion, aiming only for reversible wall motion abnormalities without perfusion imaging or left anterior descending (LAD) Doppler coronary flow reserve measurement (1). The diagnostic yield of perfusion imaging vs wall motion analysis only for CAD detection, either by adenosine cardiac magnetic resonance (aCMR) or stress echocardiography (cSE), has been tested in one of the previous studies, which confirmed that a reversible perfusion defect during pharmacologic stress has a higher predictive value for CAD detection (2). A delayed contrast appearance after microbubble destruction by high-mechanical index impulses (reduced blood flow) forms the basis for the detection of CAD using cSE.

## Case presentation

**Figure 1:**
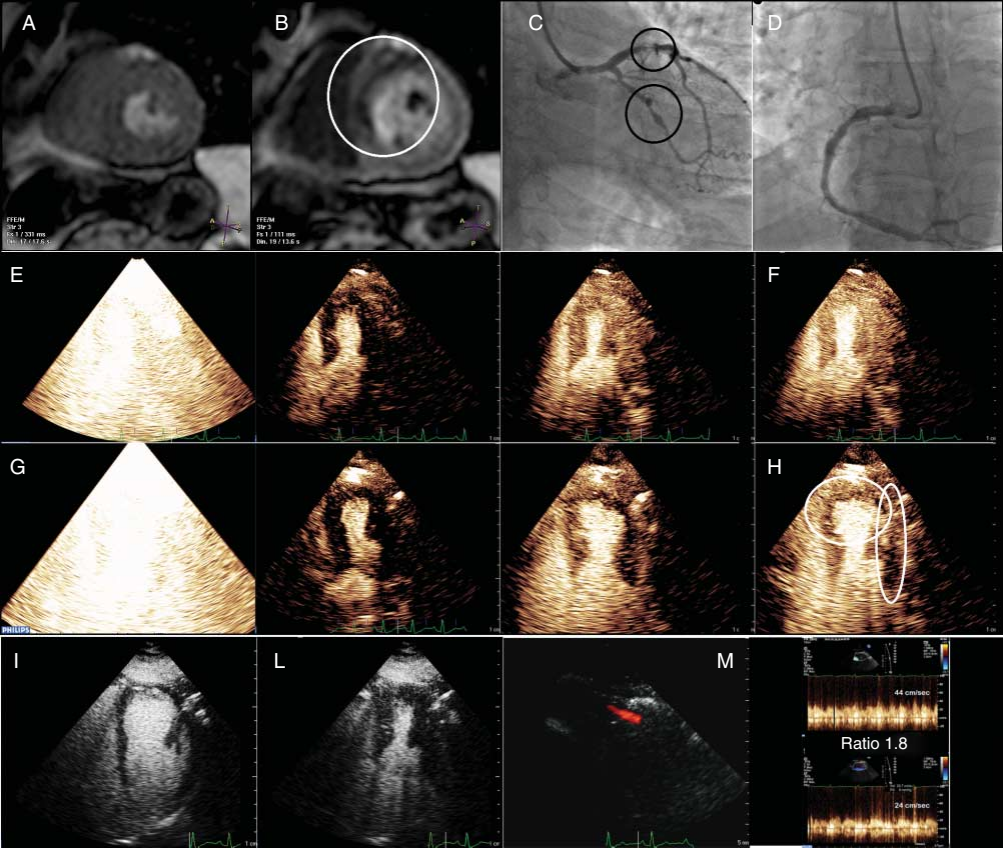
(A) aCMR rest short axis view. (B) aCMR stress short axis view. (C) Coronary angiography (LAD and circumflex arteries). (D) Coronary angiography (RCA). (E) Echocardiography: four-chamber view (flash-replenishment sequence at rest depicted from left to right). (F) Echocardiography: four-chamber view (image acquired 4 s after flash, indicating normal myocardial replenishment). (G) Perfusion defects in the lateral and apical segments after dipyridamole. (H) Still frame acquired 2 s after flash, white ovals indicate transmural lateral and subendocardial apical defects. (I) Wall motion at stress, end-diastolic image. (L) Wall motion at stress, end-systolic image. (M) Stress/rest Doppler diastolic velocity ratio on the LAD (color Doppler and pulsed-wave tracings).

A 52-year-old man presented after one episode of effort angina, normal submaximal treadmill electrocardiogram (ECG), and clearly positive aCMR for reversible perfusion defects in the LAD coronary artery territory (rest (Fig. 1A) and stress short axis view (Fig. 1B)). Coronary angiography demonstrated proximal LAD severe stenosis and mid circumflex severe stenosis (Fig. 1C, Video 1), with mild disease of the right coronary artery (Fig. 1D, Video 2). Contrast high-dose dipyridamole (0.84 mg/kg per 6 min) stress echocardiography (cSE), performed before angiography, demonstrated normal MP and wall motion at rest in the four-chamber view (flash-replenishment sequence at rest depicted from left to right (Fig. 1E, Video 3); Fig. 1F represents the image acquired 4 s after flash, indicating normal myocardial replenishment). Perfusion defects (Fig. 1G, Video 4) were shown in the lateral and apical segments after dipyridamole (Fig. 1H represents the still frame acquired 2 s after flash, white ovals indicate transmural lateral and subendocardial apical defects). Surprisingly, wall motion at stress was normal (left ventricular end diastolic image (Fig. 1I, Video 5) and left ventricular end systolic image (Fig. 1L, Video 5)) and stress/rest Doppler diastolic velocity ratio on the LAD (color Doppler and pulsed-wave tracings (Fig. 1M, Video 6)) demonstrated reduced flow reserve (44/24 cm/s, ratio=1.8).

Video 1 Coronary angiography (LAD and circumflex arteries). Download Video 1 via http://dx.doi.org/10.1530/ERP-14-0013-v1
Download Video 1

Video 2 Coronary angiography (RCA). Download Video 2 via http://dx.doi.org/10.1530/ERP-14-0013-v2
Download Video 2

Video 3 Echocardiography: four 4 chamber view (flash-replenishment sequence at rest depicted from left to right). Download Video 3 via http://dx.doi.org/10.1530/ERP-14-0013-v3
Download Video 3

Video 4 Perfusion defects in the lateral and apical segments after dipyridamole. Download Video 4 via http://dx.doi.org/10.1530/ERP-14-0013-v4
Download Video 4

Video 5 Wall motion at stress (left ventricular end diastolic image) and wall motion at stress (left ventricular end systolic image). Download Video 5 via http://dx.doi.org/10.1530/ERP-14-0013-v5
Download Video 5

Video 6 Stress/rest Doppler diastolic velocity ratio on the LAD (color Doppler and pulsed-wave tracings). Download Video 6 via http://dx.doi.org/10.1530/ERP-14-0013-v6
Download Video 6

In this case, cSE was the provocative test detecting both the LAD and circumflex lesions thanks to MP analysis, while treadmill ECG was negative and aCMR highlighted only one territory, i.e. the LAD disease. Nonetheless, wall motion during cSE resulted falsely negative in this case of three-vessel disease. Doppler coronary flow reserve, non-invasively measured on the LAD, was reduced, confirming its significant additive diagnostic role, as wall motion analysis during cSE may result falsely negative in multivessel disease and perfusion imaging is not always technically feasible.

## Investigation

See panel and linked videos showing results of coronary angiography, aCMR, and cSE.

## Discussion

Single-photon emission computed tomography using radionuclide agents is the most widely used MP technique for the assessment of CAD, although the use of first-pass contrast magnetic resonance is increasing for this purpose.

Myocardial contrast echocardiography (MCE) can evidence perfusion defects (relative decrease in contrast enhancement or prolonged time to reach contrast enhancement of one region compared with other adjacent regions). A contrast defect is usually observed first in the subendocardium and rarely extends over the full thickness of the myocardium. When analyzing loops obtained with real-time imaging, judgment of wall motion and MP is often combined. A subendocardial perfusion defect makes a wall motion abnormality much clearer and vice versa. Thus, assessment of myocardial contrast often helps by increasing the diagnostic confidence of dubious wall motion analysis. During stress echocardiography, concordant findings in wall motion and perfusion increase our confidence when assessing a dubious wall motion abnormality.

Contrast echocardiography significantly improves the image quality during rest and stress echocardiography and at the same time provides additional information on MP (3).

Reversible perfusion defects may help diagnose more comprehensively the extent and territorial distribution of functionally significant CAD, in cases of multivessel disease (4).

In the reported case, cSE with additional perfusion assessment helped diagnose CAD in the two mainly affected territories (LAD and circumflex), with confirmation by Doppler assessment of reduced flow reserve on the LAD. Unfortunately, cSE is not robust enough for ideal perfusion assessment, rarely reliably assessed in the entire left ventricle, leaving uncertainties in few segments.

aCMR has a better coverage of the whole left ventricle, but as shown in this case, it also has drawbacks that can sometimes limit its diagnostic value in multivessel disease. aCMR and adenosine MCE have been compared in one of the previous studies (5) with comparable accuracy, although MCE presents obvious logistical and cost-related advantages.

Independently from the imaging method utilized, in case of multivessel disease, the use of vasodilator stress may hamper the detection of perfusion defects in territories subtended by less severe coronary stenosis, due to relatively smaller flow reduction: aCMR is not immune to this mechanism of false-negative results. In these cases, a second imaging provocative test may be synergistic.
